# Can the prophylactic administration of tranexamic acid reduce the blood loss after robotic-assisted radical prostatectomy? Robotic Assisted Radical Prostatectomy with tranEXamic acid (RARPEX): study protocol for a randomized controlled trial

**DOI:** 10.1186/s13063-022-06447-x

**Published:** 2022-06-18

**Authors:** M. Balik, J. Kosina, P. Husek, J. Pacovsky, M. Brodak, F. Cecka

**Affiliations:** 1 Department of Urology, Charles University, Faculty of Medicine in Hradec Králové, Šimkova 870, 500 03 Hradec Kralove, Czech Republic; 2 Department of Surgery, Charles University, Faculty of Medicine in Hradec Králové, Šimkova 870, 500 03 Hradec Kralove, Czech Republic

**Keywords:** Tranexamic acid, Robotic-assisted radical prostatectomy, Bleeding prophylaxis

## Abstract

**Background:**

The prophylactic administration of tranexamic acid reduces blood loss during procedures at high risk of perioperative bleeding. Several studies in cardiac surgery and orthopedics confirmed this finding. The aim of this prospective, double-blind, randomized study is to evaluate the effect of tranexamic acid on peri-and postoperative blood loss and on the incidence and severity of complications.

**Methods/design:**

Based on the results of our pilot study, we decided to conduct this prospective, double-blind, randomized trial to confirm the preliminary data. The primary endpoint is to analyze the effect of tranexamic acid on perioperative and postoperative blood loss (decrease in hemoglobin levels) in robotic-assisted radical prostatectomy. The additional endpoint is to analyze the effect of tranexamic acid on postoperative complications and confirm the safety of tranexamic acid in robotic-assisted radical prostatectomy.

**Discussion:**

No study to date has tested the prophylactic administration of tranexamic acid at the beginning of robotic-assisted radical prostatectomy. This study is designed to answer the question of whether the administration of tranexamic acid might lower the blood loss after the procedure or increase the rate and severity of complications.

**Trial registration:**

ClinicalTrials.gov NCT04319614. Registered on 25 March 2020

## Background

Prostate adenocarcinoma is the second most common malignancy in men. The incidence increases over time and with patient age. It is the second most common cause of death due to malignancy in men, after lung cancer. Standard treatment includes radical prostatectomy or radiotherapy in patients with life expectancy more than 10 years (LE1b, GRA) [1–3].

In recent years, there is a general tendency towards minimally invasive surgical procedures. In the treatment of localized prostate cancer, laparoscopic or robotic-assisted radical prostatectomy has become the standard treatment method. Despite tremendous development in the technology and technique of robotic-assisted radical prostatectomy (RARP) over more than 25 years, new ways and methods to improve oncological and functional outcomes are still needed [4–8].

Intraoperative blood loss is one of the most used parameters to assess the quality of surgical procedures. Rothermel et al. demonstrated that visual estimation of operative blood loss is unreliable and inaccurate [9]. Poletajev et al. concluded that blood loss during laparoscopic radical prostatectomy (LRP), which is considered comparable to the RARP, is clinically insignificant but can reach up to 600 ml, according to the laboratory findings [10].

Decreasing peri- and postoperative blood loss may lead to faster recovery after surgical procedures [11]. Sampaio et al. found five randomized prospective studies evaluating 838 patients with antifibrinolytics in cancer surgery. This meta-analysis found no evidence that antifibrinolytics increase the risk of thromboembolic complications. The authors have shown that it is effective in reducing total perioperative blood loss and also in the need for blood transfusion [12]. Several hemostyptic agents have been tested in this setting [13]. Hemostatic recombinant activated factor VII was originally developed to manage uncontrolled bleeding in hemophiliac patients. It has been used off label in cardiac surgery, neurosurgery, hepatic surgery, and open radical prostatectomy. Its use was associated with a higher risk of arterial thromboembolic events, especially in older patients, and therefore should not be used in prophylactic setting in order to reduce blood loss. Its high price disqualifies it from the routine use in urological procedures [14]. Antifibrinolytic aprotinin is a broad-spectrum inhibitor of serine proteases, including those in the coagulation cascade. It has been shown to have a better effect on the prevention of peri- and postoperative bleeding than tranexamic acid. However, in 2008, aprotinin was withdrawn from the market due to massive side effects, including increased perioperative mortality during complex cardiac surgery [15].

Tranexamic acid is an antifibrinolytic agent used to relieve bleeding. The mechanism of action lies in binding to plasma-free plasminogen with higher affinity than tissue plasminogen activator. It prevents its conversion to plasmin, which is responsible for the degradation of fibrin polymers. It results in greater stability of the fibrin clot at the site of bleeding, and therefore, it lowers blood loss [16–18]. The use of tranexamic acid during or after the procedure does not improve results, unlike administration prior to surgery. A biological explanation is that tranexamic acid may bind plasminogen in the early phase of the fibrinolytic cascade, after the beginning of the procedure, reducing tissue plasminogen activator activity up to 80% [19].

In urology, increased conversion of plasminogen to plasmin should occur, both by washing the tissue plasminogen activator from the destroyed tissue and by urokinase present in the urine [20]. Several studies did not confirm the positive effect of tranexamic acid in terms of reduced perioperative and postoperative blood loss in prostate transurethral resection [21, 22]. Other studies showed the positive effect in transurethral prostate resection [23, 24], open radical prostatectomy [25], and open radical cystectomy [26], including a recent meta-analyses [27, 28]. Due to the limited number of studies and the high heterogeneity of the results, more trials with a large number of patients are necessary to confirm these findings [28].

No study to date has tested the prophylactic administration of tranexamic acid at the beginning of robotic-assisted radical prostatectomy in a prospective manner. A Cochrane review concluded that randomized controlled trials different from cardiac surgery are needed to assess the efficacy and safety of tranexamic acid in surgical procedures [13].

## Methods/design

The RARPEX trial is designed as a randomized, placebo controlled, surgeon- and patient-blinded superiority single-center study with two parallel groups. Randomization will be performed as a block randomization with 1:1 allocation.

### Objectives and hypothesis

The primary aim of the Robotic **A**ssisted Radical Prostatectomy with EXacyl (RARPEX) trial is to investigate the effect of tranexamic acid on perioperative and postoperative blood loss (decrease in hemoglobin levels) in robotic-assisted radical prostatectomy.

H0: The drop of hemoglobin level after the procedure is similar in both groups.

HA: The drop of hemoglobin level after the procedure in control group is higher than in study group.

Additional endpoint of the RARPEX trial is to analyze the effect of tranexamic acid on postoperative complications and confirm the safety of tranexamic acid in robotic assisted radical prostatectomy.

### Study population and eligibility criteria

All patients who are scheduled for operation due to low or intermediate risk prostate cancer in our institution will be screened and assessed for eligibility.

Only patients who will undergo robotic-assisted radical prostatectomy with suturing of dorsal complex vein (DVC) bundle at the beginning of the procedure without pelvic lymph node dissection will be included in the study. Patients with non-standard procedures or a procedure associated with higher morbidity will be excluded from the study to achieve a homogeneous study group.

Detailed inclusion and exclusion criteria are described below.

The following are the inclusion criteria:

1. Patient scheduled for robotic-assisted radical prostatectomy without pelvic lymph node dissection
2. Signed informed consent provided
3. Body mass index ≤ 35
4. Age of patient ≤ 75 years
5. Operating surgeon with experience of more than 100 cases

The following are the exclusion criteria:

1. Body mass index > 35
2. Age of the patient > 75 years
3. Coagulation disorder (congenital or iatrogenic due to the chronic use of anticoagulants)
4. Thromboembolic, cerebral, or an acute coronary event within the 6 months prior to prostatectomy
5. Chronic renal insufficiency (arbitrary cutoff level of creatinine 200 μmol/l)
6. Allergic reaction to tranexamic acid
7. Operating surgeon with experience < 100 cases
8. Patient participating in another study

If subjects do not meet the inclusion criteria or withdraw their consent, they will be excluded from the study. The researcher will record the reason for their withdrawal.

#### Criteria for discontinuing

A trial participant should terminate intervention if they wish to do so or if the investigator judges it necessary due to any of the following reasons:

- Withdrawal of informed consent
- Patient refusal or non-compliance to the protocol
- Occurrence of an unexpected serious adverse effect
- Non-standard procedure (non-sutured dorsal vein complex in the beginning of the procedure, rectal, small bowel, or ureteral wall injury)
- Other hemostyptic drug or material used during or after the procedure

### Sample size calculation

The sample size calculation is based on the data from our pilot study [29]. We used two-sample *T* tests allowing unequal variance with respect to the primary endpoint, which is the difference in hemoglobin level drop on postoperative day (POD) 1. The level of significance is set to 5 g. With *α* = 1% and *β* = 10%, a sample size of 64 patients per group is necessary to detect a clinically significant difference between the groups. With an expected dropout rate over 33%, we plan to enroll 200 patients in the study.

### Ethics, study registration, and consent

This trial was approved by the independent ethics committee at the University Hospital Hradec Kralove (registration number 201903 I90P). The RARPEX trial will be conducted in the context of Good Clinical Practice and in accordance with the Declaration of Helsinki. The trial is registered at ClinicalTrials.gov under the registration number NCT04319614. All patients who are scheduled for robotic-assisted radical prostatectomy in our institutions will be screened for eligibility and informed in detail about the RARPEX trial. Informed consent will be obtained from each participant. The study procedures, risks, benefits, and data management will be clarified with the patients before they are asked to give their informed consent to participate. Any participant in this study may withdraw consent or voluntarily cease to participate at any time for any reason.

### Assignment of interventions

#### Allocation

##### Sequence generation

Participants will be randomly allocated to one of the groups before the surgical procedure after meeting the eligibility criteria with a ratio of 1:1. Randomization will be accomplished using random block sizes without stratification (block randomization) using statistical software approved for this purpose, e.g., from the website www.randomization.com. The goal is to obtain homogeneity between the groups.

##### Concealment mechanism

Opaque, sealed envelopes will be produced, labeled with the randomization number and containing a sheet that states the group allocation for the patient. Randomization envelopes will be used in consecutive order. Basic characteristics of the patient and the day of randomization will be documented on a data sheet so that compliance with the randomization scheme may be checked retrospectively. To maintain the double-blinding, the placebo and active ingredient are identical and cannot be distinguished by the appearance of the participants or staff. The code assigned to subjects will be kept sealed and will not be released until the end of the clinical trial. Cases in which the blinding must be unsealed, such as a serious adverse drug reaction, will be managed using a separate envelope created for each subject so that only their randomization is revealed. Randomization and blinding will not be revealed to the researchers until the end of the study. If patients are excluded from the study after randomization, their numbers will not be reused.

##### Implementation

Unblinded nurse will prepare an infusion set according to the information in the sealed envelope with the patient’s study number on the day of the procedure. For patients in the intervention group 1, the dose of tranexamic acid according to target 20 mg/kg will be added to 100 ml of physiological saline. For patients in the intervention group 2, she will add no other substance to 100 ml of physiological saline. Infusion will be given into the sealed envelope, sent to the operating theater, and given within 5 min after the robotic system is docked.

#### Blinding

The double-blind randomized experimental scheme of the study allows to limit the statistical bias as well as the intervention bias. So, active treatment (tranexamic acid) and placebo will be allocated in blind: neither the patient nor the operating surgeons, attending physicians, nursing staff, and outcome assessors will know the allocated treatment. Furthermore, the presentation and the packaging of active treatments and placebos will be identical.

#### Unblinding

The unblinding will be carried out systematically at the end of the study. The study team members and healthcare providers do not have access to the treatment allocation code. However, if an investigator wants to introduce a medication that should be not taken at the same time to the study treatment, the blinding code will be broken. No expected adverse event in the study will require an emergency unblinding. In case of suspected unexpected serious adverse reaction, the sponsor will declare the serious and unexpected adverse reaction to the health.

### Study treatment

Based on the literature [16–18], and our pilot study [29], we decided to administer a single dose of tranexamic acid, corresponding to 20 mg/kg in 100 ml saline to all patients in the treatment group 1 at the beginning of the procedure. In the control group 2, we administer only 100 ml saline as placebo.

For prophylaxis of venous thromboembolism (according to the rules of our institution), the combination of mechanical device (graduated compression stockings) and pharmacologic agents (low-molecular weight heparin (LMWH)) in both treatment groups will be used. The prophylactic dose of LMWH is administered in the evening before the procedure, and the next dose at least 8 h after the procedure and then every evening at least till POD 7. Antibiotic prophylaxis is provided by a single dose of potent aminopenicillin as recommended by the antibiotic center: fluoroquinolone in patients with an allergy to aminopenicillin. During the procedure, the console time and weight of the prostate are monitored. Relevant concomitant care and interventions are permitted if necessary.

The surgical technique is standardized and has been described previously [30]. Standard robotic-assisted radical prostatectomy without pelvic lymphadenectomy using DaVinci Xi surgical system is performed. The dorsal vein complex (DVC) is sutured at the beginning of the procedure with two rounds of resorbable monofilament suture. To accelerate the return of continence, a modified Rocco stitch is performed in all patients. The anastomosis is performed by two tied V-loc stitches. No additional manipulation, such as fibrin glue or reinforcement with meshes, is allowed. Patients with rectal or bowel injury during the procedure will be excluded from evaluation.

For 3 h after the procedure, patients of both groups will stay in the intermediate care unit; after moving to the standard ward, blood samples will be obtained. On POD 1, all the patients will start with mobilization and solid food intake. The volume of the fluids in the drain will be measured on postoperative day 1, and if the volume will not exceed 200 ml for 24 h, the drain will be extracted. If the volume is higher than 200 ml/24 h and the creatinine level in drain fluid exceeds 500 μmol/l, urinary leakage will be confirmed [31]. On POD 2, the patients will be released for home care with an indwelling permanent urinary catheter. On POD 7, the urinary catheter and skin sutures will be extracted, and the blood sampling and ultrasound of the lower abdomen will be performed. Three months after the procedure, a follow-up visit is scheduled. The evidence of complication and level of prostatic specific antigen (PSA) is monitored.

Upon completion in 200 patients, the statistical processing will be performed, and patients will be unblinded.

### Safety aspects

Robotic-assisted radical prostatectomy is a highly technically demanding procedure. High-volume surgeons with great experience have better results than low-volume surgeons with less experience [32].

To avoid bias based on the learning curve of the surgeons, every surgical procedure will be performed by a senior surgeon who has experience with at least 100 robotic-assisted radical prostatectomies. Administration of tranexamic acid at the beginning of the procedure by an anesthesiologist is a simple common procedure, performed on a routine basis, and no special training is necessary, and no complications are expected.

### Data collection

A daily visit of the study patients will be made by clinical investigators or a delegated physician. All protocol-required information collected during the trial will be entered into the patient’s record form.

Patient identification on the case report form (CRF) is through their unique trial identifier, allocated at the time of recruitment. Data coding will be carried out at various stages during the study. The patient’s record form is subject to audit by the principal investigator (PI) and Institutional Review Board (IRB).

Preoperative data gathered include patient age, body mass index, American Society of Anesthesiologists physical status classification system score, and comorbidities. Intraoperative data to be collected include surgery duration (skin to skin), console time (console surgeon activity time), and weight of the prostate.

Laboratory tests will include blood cell count (BCC), hematocrit test, and plasmatic creatinine level at the beginning of the procedure, 3–6 h after the procedure, on POD 1, POD 2, and POD 7 in the morning.

The differences between the hematocrit and hemoglobin levels (eventually weighted for the grams of the prostatic tissue) and hemoglobin/creatinine ratios will be obtained.

The volume of the fluids in the drain will be measured on postoperative day 1, and if the volume does not exceed 200 ml for 24 h, the drain is extracted. If the volume will be higher than 200 ml/24 h and the creatinine level in the drain fluid exceeds 500 μmol/l, urinary leakage will be confirmed (29). Patients with confirmed urinary leakage or urinoma will be excluded from the evaluation.

Postoperative course assessments will include duration of intermediate/intensive care, hospital stay including readmissions for postoperative complications, reinterventions (reoperations, endoscopic and interventional radiology procedures), the reasons for readmissions, and transfusion rates. The patients will be seen by a clinical investigator 3 months after the surgery in an outpatient fashion. The evidence of complication and level of prostatic specific antigen (PSA) will be monitored. Upon completion of 200 patients, the statistical processing will be performed, and results will be unblinded.

All study-related information will be stored securely at the study site. All participant information will be stored in locked file cabinets in areas with limited access. All reports, data collections, processes, and administrative forms will be identified by a coded ID number only to maintain participant confidentiality. All records that contain names or other personal identifiers will be stored separately from study records identified by code number.

### Data monitoring

The trial will be monitored by the principal investigator (PI) and Institutional Review Board (IRB) without additional monitoring by an Independent Safety Monitor or Data Safety Monitoring Board. The patient’s CRF is a subject to audit by the PI and IRB. Data integrity and study credibility depend on factors such as ensuring adherence to the protocol and using quality control measures to establish and maintain high standards for data quality. To ensure that accurate, complete, and reliable data are collected. The PI provides training to the site staff in the format of investigator meetings. The data are validated, and discrepancy reports are generated following data entry to identify discrepancies, such as out-of-range values, inconsistencies, or protocol deviations based on data validation checks. The PI will be actively involved in reviewing the progress of each participant on protocol and will report, to the IRB, adverse events and unexpected problems that may influence the IRB’s decision to allow the trial to continue (in accordance with the IRB’s policies). The IRB is convened to carry out reviews of the data at staged intervals during the study. The dropout rate is expected to be negligible with little or no missing data for the primary outcome measure. Standard approaches will be used to detect patterns in missing data for the other outcomes. Monitoring activities and changes to the study emanating from the monitoring activities will be described in the annual progress report.

#### Adverse event reporting and harms

Adverse events (AE) reported by the patient or by the investigator will be recorded and scored according to the NCI Common Terminology Criteria for Adverse Events (CTCAE). If AE happen during the experimental treatment period, all the details will be notified in the CRF as the time of occurrence, clinical symptoms and signs, degree, duration, and causal relationship with the treatment. In case of serious adverse events (SAE), the investigator has to inform the PI within 2 h after finding the SAE at the latest. The investigator may stop immediately the treatment if it is considered in the best interest of the patient. The imputability of the SAE with the experimental must be established. SAE with a doubtful, possible, probable, or highly probable consequence of tranexamic acid administration will be considered as associated to it. If SAE are unexpected, they will be designed as suspected serious adverse reactions (SUSARs). In this case, the PI has to declare it to the ethical committee and IRB. This must occur not later than 15 days after the first knowledge of the SAE. In case of fatal or self-threatening cases, the delay will be reduced to a maximum of 7 days and 8 days supplementary for the report completion. Once a year, the PI will create a report with the complete list of SAE that could be associated to the experimental medication including expected and unexpected effects, and a precise and critical analysis of the safety of participants included in the study.

The IRB of the study will systematically meet every 12 months after the beginning of the trial and will decide whether or not to continue the study.

#### Ancillary and post-trial care

Standard care is provided after participants have finished the study. All adverse events or reactions will be followed until stabilized or resolved.

#### Interim analyses

No interim analysis has been planned for this study.

#### Protocol amendments

All the modifications needing substantial amendments will be discussed within the IRB and ethical committee. The modifications must be accepted by these instances. Once accepted, modifications will be notified in trial registries and documents. Patients included in the study will be informed on important protocol modifications if personally relevant to them.

### Primary and secondary endpoints

The primary endpoint is to analyze the effect of tranexamic acid on perioperative and postoperative blood loss in a randomized and prospective manner. A clinically significant difference between the groups on POD 1 is set to 10 g/l. The sample size was calculated for this setting.

The additional aim of the RARPEX trial is to analyze the effect of tranexamic acid on other postoperative complications: wound infection, intraabdominal collections, urinary leakage, delayed gastro-intestinal emptying, postoperative hemorrhage, pneumonia, and abdominal rupture, especially analyzing the incidence of cardiac events and venous thromboembolism (VTE) after the procedure (Table 1).

**Table 1:** Clinical parameters and postoperative complications for analysis

Parameters	Definitions
Hospital stay	Days from initial operation to hospital discharge plus any readmission within 30 days
Console time	Time of console surgeon activity (min)
Postoperative hemorrhage	Evidence of blood loss from drains, based on ultrasonography or CT
Transfusion rate	The number of blood transfusions
Urinary leakage	Evidence of creatinine level > 500 μmol/l and volume of the drain output exceeds 200 ml/24 h, confirmed on cystography
Lymphorrhea	Evidence of creatinine level < 500 μmol/l, hematocrit < 0.2, and volume of the drain output exceeding 200 ml/24 h, no urinary leakage on cystography
Intraabdominal fluid collection	Collection of fluid measuring ≥ 3 cm associated with clinical or laboratory abnormalities
Symptomatic fluidothorax	Fluid in the pleural cavity associated with respiratory distress or a need to evacuate the fluid
Thromboembolism	Unilateral limb swelling, acute respiratory insufficiency, based on ultrasonography or CT
Myocardial infarction	Increase of serum concentration of CK-MB and troponin and/or the following ECG changes: new Q waves ≥ 0.04 in duration, new persistent ST elevation, and/or depression
Brain stroke	Presence of neurological symptoms, findings on CT scan or MRI
Pneumonia	Presence of a new infiltrate on chest X-ray, as well as the following: body temperature > 38 °C, abnormal elevation of WBC, or positive sputum, and requiring antibiotic treatment
Acute renal failure	Serum creatinine > 300 μmol/l and/or need for dialysis
Wound infection	Surgical site infection associated with laparotomy that develops during the initial hospital stay
Urinary tract infection	Culture-positive urine, pyuria, or bacteriuria on urinalysis requiring antibiotic treatment

*CK-MB* creatine kinase MB isoenzyme, *ECG* electrocardiogram, *WBC* white blood cells, *CT* computer tomography, *MRI* magnetic resonance imaging

Postoperative complications are graded based on severity according to the Clavien-Dindo definition (Table 2) [33].

**Table 2:** Complication grades according to the Dindo-Clavien classification scheme

Grade	Definition
Grade I	Any deviation from the normal postoperative course without the need for pharmacological treatment or surgical, endoscopic, and radiologic intervention
Grade II	Requiring pharmacological treatment with drugs other than those allowed for grade I complications
Grade III	Requiring surgical, endoscopic, or radiological intervention
Grade IIIa	Intervention not under general anesthesia
Grade IIIb	Intervention under general anesthesia
Grade IV	Life-threatening complications requiring intensive care unit management
Grade IVa	Single-organ dysfunction
Grade IVb	Multiorgan dysfunction
Grade V	Death of the patient

We plan to seek for adverse events with repetitive blood sampling and imaging to prevent the underreported frequency of these events. But we are aware that the incidence is low, and our data will might not have sufficient power to make a definitive conclusion about safety.

## Methods for avoiding bias

### Minimizing systemic bias

**Fig. 1:**
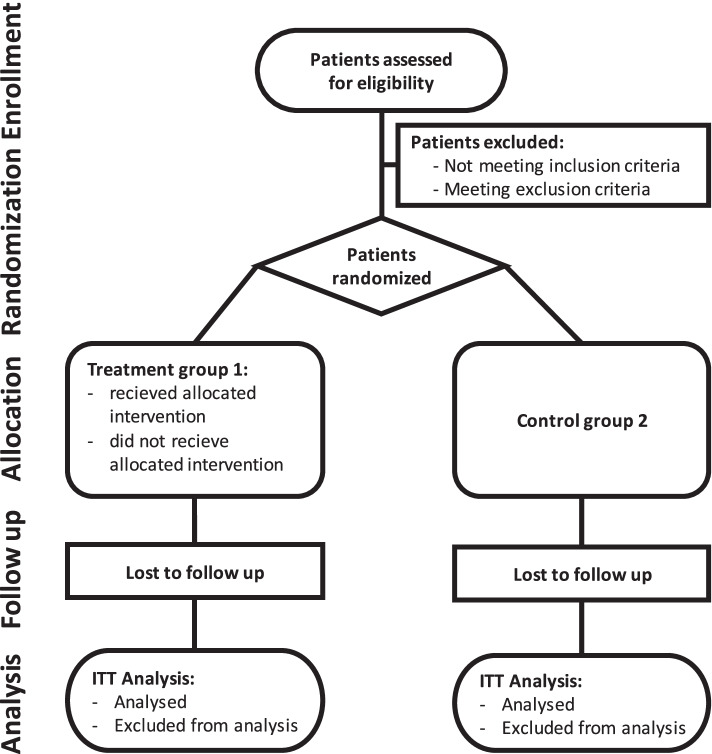
Process phases flowchart of randomized trial according to the CONSORT guidelines

Participants will be randomly allocated to one of the groups before the surgical procedure after meeting the eligibility criteria with a ratio of 1:1. Operating surgeons, attending physicians, nursing staff, and outcome assessors will be blinded. The randomization process will follow the CONSORT guidelines (Fig. 1) [34].

**Fig. 2:**
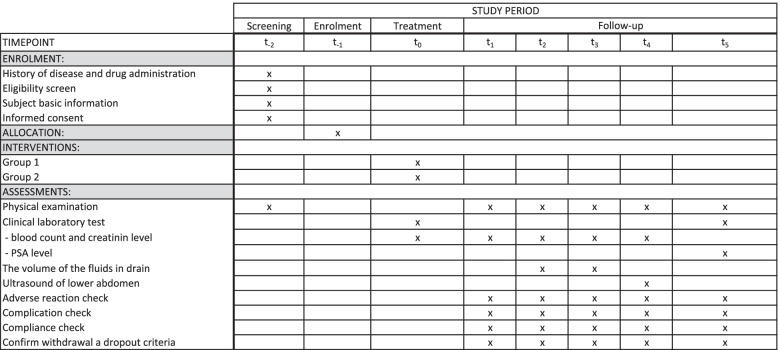
The schedule of enrollment, interventions, and assessments of randomized trial according to the SPIRIT guidelines (*t*_−2_ = − 14 − 1 days; *t*_−1_ = − 1 day; *t*_0_ = at the beginning of the procedure; *t*_1_ = 3 h after the procedure; *t*_2_ = POD1; *t*_3_ = POD2; *t*_4_ = POD7; *t*_5_ = 3 months after the procedure ± 7 days)

Standard Protocol Items: Recommendations for Interventional Trials (SPIRIT) 2013 Checklist was used to improve the quality of the study protocol [35] (Fig. 2).

### Minimizing treatment bias

Administration of tranexamic acid at the beginning of the procedure by an anesthesiologist is a simple common procedure, performed on a routine basis, which eliminates a learning curve.

All patients will undergo robotic-assisted radical prostatectomy without pelvic lymphadenectomy using the same technique. All surgeons participating in the study are familiar with this procedure. The dorsal vein complex (DVC) will be sutured at the beginning of the procedure.

### Minimizing measurement bias

Measurement of hemoglobin level drop and detection and grading of postoperative complications will be based on data in the patient’s record form. Patient and clinical investigators or a delegated physician will be blinded.

## Statistical methods

Each patient’s allocation to the analyzed population will be defined prior to the analysis and will be documented. In the full analysis set, patients will be analyzed as randomized according to the intention-to-treat principle. The intention-to-treat principle implies that the analysis includes all randomized patients. The per-protocol analysis set will include all the patients without major protocol deviation. Deviations from the protocol will be assessed as major or minor. Patients with major deviations from the protocol will be excluded from the protocol analysis. The safety analysis set will analyze the patients according to the treatment. The null hypothesis assumes that there is no difference in the hemoglobin level drop after the procedure in both groups. We will use a two-sample *T* tests allowing unequal variance with respect to the endpoint, which is the drop of hemoglobin levels. Differences between age and PSA will be assessed by the non-parametric Mann-Whitney *U* test. BMI and specimen weight will be compared by the Kolmogorov-Smirnov test. A *P*-value < 0.05 will be considered statistically significant. Statistical analyses will be performed using the NCSS statistical software (NCSS, Kaysville, UT, USA).

### Health economic analysis

A cost analysis will be performed in order to compare the cost per patient in each treatment group. Data relating to patients’ primary hospital admission is collected prospectively until primary hospital discharge (including but not limited to their length of stay on a ward and in the intensive care unit). Data relating to readmissions, accident and emergency attendances, and outpatient attendances will be collected at the *t*_5_ visit. Unit costs will be obtained from public sources, e.g., Department of Health reference costs. The differences in costs between the groups will be estimated using regression methods adjusted for baseline and other covariates where appropriate.

## Discussion

### Blood loss

One of the greatest risks of any technical demanding surgical procedure is bleeding. It is similar to radical prostatectomy. The robot-assisted approach leads to a significant reduction of blood loss. Nevertheless, efforts to reduce the blood loss even more are eagerly awaited. Perioperative hemorrhage makes the surgical terrain unclear, makes it difficult to dissect the tissue precisely, increases the risk of complications, and worsens functional and oncological results. Excellent experience in orthopedics and other cardio-surgical fields with the prophylactic administration of tranexamic acid after the introduction to anesthesia gives rise to the hope of successful use in urology as well.

We decided not to estimate perioperative blood loss visually, because of its inaccuracy and unreliability documented by Rothermel et al. [9]. The cause lies in many biases as we confirmed in our pilot study [29]. The volume of suction fluid is affected by urine coming out of the open urinary tract with different intensities for various lengths of time and by variable lymphatic secretion from damaged tissue.

Therefore, we focused on the drop in hemoglobin levels. Chesnut et al. in their retrospective evaluation of 3631 patients found a median decrease in hemoglobin 4 h after the procedure by 11 g/l and 14 h after the procedure by 20 g/l [36]. In our pilot study, we found average hemoglobin level drop 3 h after the procedure by 9.3 g/l vs. 17.3 g/l. On POD 1, it was 17.6 g/l vs. 25.6 g/l in favor of the study group. The average difference between the study and control groups was 8 g/l in both measurements if the dorsal vein complex was sutured at the beginning of the procedure. We considered the difference to be promising and set the level of significance in sample size calculations to 10 g/l.

A decrease in hemoglobin level by 30% or 40 g/l or need for blood transfusion was reported in the literature as clinically significant blood loss [37]. Considering these data, the difference of 10 g/l may seem insufficient. However, on the other hand, a similar change in hemoglobin level was found after administration of one unit of packed red blood cells [38, 39]. The estimated blood loss is a significant measurement of quality of care even if the patient receives no blood transfusion.

A sufficient number of blood counts will be analyzed in order to acquire sufficient data. Meunier et al. described a decrease in hemoglobin level after a single major blood loss (due to the blood donation) even after 6 days [40]. Prospective data are also needed to answer questions about the timing and frequency of sampling after RARP [36].

Chan et al. proved that the results can be significantly affected by the weight of the prostate. A larger prostate could mean longer operating time and greater blood loss. Therefore, the results will be weighted for the grams of the prostatic tissue [41].

### Dose

The therapeutic concentration of tranexamic acid in plasma ranges from 5 to 10 mg/kg. After an intravenous dose of 10 mg/kg, plasma concentration was maintained for 3 h, but orthopedics proved to be inadequate. Based on the abovementioned literature, we decided to administer a single dose, corresponding to 20 mg/kg to all patients in the treatment group 1 at the beginning of the procedure.

### Complications

Although transfusion rates after open retropubic radical prostatectomy (RRP) are low - 3.4%, and robotic approach dropped the rate even lower to 0.8% [42], in our pilot study to 1.0% [29]. The safety of homologous transfusion has improved over time, but the possibility of having transfusion-related reactions or acquiring transfusion-transmitted diseases still remains [43]. Concerns have been raised about the possible relationship between the administration of blood derivatives and an increased risk of relapse of malignancy and tumor-specific mortality [44, 45]. At this moment, we are aware of only one retrospective study showing a poor correlation between postoperative hemoglobin assessment and transfusion requirement among patients undergoing minimally invasive radical prostatectomy [36]. Prospective data is lacking.

Radical prostatectomy is associated with a higher risk of thromboembolism. Open radical prostatectomy has a considerably higher risk of thromboembolic events (1.0—15.7%) compared to robotic (0.2–3.7%) and laparoscopic (0.4–6.0%) approaches [46]. Administration of antifibrinolytics, which potentially increase the risk of thromboembolism in laparoscopic surgery for pelvic malignancy, may seem too risky. According to the literature data from recent meta-analyses, no demonstration of an increased risk of thromboembolism following treatment with tranexamic acid was observed [27, 28].

## Conclusion

Despite the enormous development in robot-assisted radical prostatectomy over 25 years, improvement is still needed. One possibility is to implement the ERAS (early recovery after surgery) protocol in everyday practice. Each of the original 22 recommendations (for example, preoperative nutritional examination and nutritional preparation, intestinal preparation, fasting time, prevention of thromboembolism, antibiotic prophylaxis, decolonization of the skin, minimally invasive approach, prevention of hypothermia, intestinal prokinetics, etc.) does not significantly improve the postoperative results alone. The prophylactic use of tranexamic acid at the beginning of robotic-assisted prostatectomy could be another piece of this mosaic. Because strategies that lead to small improvements might be meaningful from a system perspective if they can be delivered easily, at low cost, and at a population level ([47].).

## Trial status

The RARPEX trial is recruiting patients under protocol version 1.0 since February 24, 2020. The last patient is expected to be recruited by February 1, 2022.

## Data Availability

The materials described in the manuscript, including all relevant raw data, will be freely available to any scientist wishing to use them for non-commercial purposes, without breaching participant confidentiality. The datasets used and analyzed during the current study will be available from the corresponding author on reasonable request. In the event of publications arising from such analyses, those responsible are required to provide the principal investigator with a copy of any intended manuscript for approval prior to submission. Dissemination policy: It is anticipated that the study findings will be published in national and international peer-reviewed journals and presented at national and international meetings and to appropriate patient groups, all of which will be led by the PI. Upon request, participants may receive a lay summary of the principal study findings. The most significant results will be communicated to the public through press releases, and a paper on the primary outcome and safety data will be published soon after analysis and review. An ongoing update of the trial is maintained on the website ClinicalTrials.gov.

## Abbreviations

AE: Adverse events
CRF: Case record form
BCC: Blood cell count
CRA: Clinical research associate;
CTCAE: Common Terminology Criteria for Adverse Events
CT scan: Computed tomography scan
DVT: Deep vein thrombosis
IRB: Institutional Review Board
LMWH: Low-molecular weight heparin
LRP: Laparoscopic radical prostatectomy
MRI: Magnetic resonance imaging
PE: Pulmonary embolism
PI: Principal investigator
POD: Postoperative day
RARP: Robot-assisted radical prostatectomy
RRP: Open retropubic radical prostatectomy
SAE: Serious adverse events
SUSAR: Suspected serious adverse reaction
VTE: Venous thromboembolism
